# Detection of Foot-and-Mouth Disease Virus in Swine Meat Juice

**DOI:** 10.3390/pathogens9060424

**Published:** 2020-05-29

**Authors:** Sean Yeo, Ming Yang, Martin Nyachoti, Rolf Rauh, Johnny D. Callahan, Charles Nfon

**Affiliations:** 1 National Centre for Foreign Animal Disease, Canadian Food Inspection Agency, 1015 Arlington Street, Winnipeg, MB R3E 3R2, Canada; sean.yeo@canada.ca (S.Y.); ming.yang@canada.ca (M.Y.); 2 Department of Animal Science, Faculty of Agricultural and Food Sciences, University of Manitoba, Winnipeg, MB R3T 2N2, Canada; Martin.Nyachoti@umanitoba.ca; 3 Tetracore, Inc., 9901 Belward Campus Drive, Suite 300, Rockville, MD 20850, USA; rrauh@tetracore.com (R.R.); jcallahan@tetracore.com (J.D.C.)

**Keywords:** skeletal muscle, meat juice, foot-and-mouth disease, FMDV RNA, FMDV antigen

## Abstract

Foot-and-mouth disease virus (FMDV) is a highly contagious agent that impacts livestock industries worldwide, leading to significant financial loss. Its impact can be avoided or minimized if the virus is detected early. FMDV detection relies on vesicular fluid, epithelial tags, swabs, serum, and other sample types from live animals. These samples might not always be available, necessitating the use of alternative sample types. Meat juice (MJ), collected after freeze-thaw cycles of skeletal muscle, is a potential sample type for FMDV detection, especially when meat is illegally imported. We have performed experiments to evaluate the suitability of MJ for FMDV detection. MJ was collected from pigs that were experimentally infected with FMDV. Ribonucleic acid (RNA) was extracted from MJ, sera, oral swabs, and lymph nodes from the same animals and tested for FMDV by real-time reverse transcription polymerase chain reaction (rRT-PCR). MJ was also tested for FMDV antigen by Lateral Flow Immunoassay (LFI). FMDV RNA was detected in MJ by rRT-PCR starting at one day post infection (DPI) and as late as 21 DPI. In contrast, FMDV RNA was detected in sera at 1–7 DPI. Antigen was also detected in MJ at 1–9 DPI by LFI. Live virus was not isolated directly from MJ, but was recovered from the viral genome by transfection into susceptible cells. The data show that MJ is a good sample type for FMDV detection.

## 1. Introduction

Foot-and-Mouth Disease (FMD) is a condition caused by foot-and-mouth disease virus (FMDV) that affects cloven-hoofed animals such as cattle, pigs, and sheep [1]. FMDV is a ribonucleic acid (RNA) virus that is highly contagious among susceptible animals and produces symptoms which include fever, reduced appetite, or vesicular lesions [1]. Mortality rates of animals infected by FMDV are generally low among adults, but very high in neonates [1]. Animals infected with FMDV have greatly reduced milk production, decreased draught power, and lose significant weight [1,2]. FMDV remains endemic in several countries around the world and impacts their respective agricultural industries [3]. Countries that have obtained FMDV-free designation by the World Organization for Animal Health (OIE) always face the risk of potential outbreaks. These FMDV-free countries utilize preventative measures to minimize the risk of an outbreak. Animals suspected of having a vesicular disease are tested to ensure the absence of FMDV as the clinical signs are indistinguishable from its differentials [2]. Procedures recommended by the OIE to identify FMDV are antigen enzyme-linked immunosorbent assays (Ag ELISAs), virus isolation (VI), real-time reverse transcription polymerase chain reactions (rRT-PCRs), or pen-side tests such as lateral flow immunoassays (LFI). These tests require samples such as vesicular fluid, epithelial tags, blood, milk, and swabs of the nares, oral cavity, or lesions, but these are not always accessible. Meat juice (MJ) is a potential alternative that can be used in situations where blood cannot be collected due to animal death or when the entire carcass is absent. This is common in the case of illegally imported meat into countries free of FMDV. Illegally imported meat contaminated with FMDV can potentially infect domestic farm animals and induce an outbreak. The 2001 UK FMDV outbreak was attributed to a single farm that fed untreated waste to pigs [4]. This epidemic resulted in culling of millions of animals to contain the virus and prevent further spreading [5].

MJ is a liquid transudate that can be collected from tissues after a freeze-thaw cycle. The cycling allows formation of ice crystals to disrupt cell membrane integrity and subsequent release of the intracellular contents, serum, and lymphatic fluid into the external environment [6]. Success has been demonstrated previously with MJ for the detection of various viruses by rRT-PCRs and ELISAs. Both assays have proven successful for detecting porcine reproductive and respiratory syndrome virus (PRRSV) in MJ from infected animals [7]. The RT-PCR detects the viral genome, while the ELISA detects antibodies produced by the animal in response to the virus. Virus detection with PCR was also successful for MJ samples containing African swine fever virus [8]. Other pathogens have also been detected by measuring the antibody response against hepatitis E virus [9], Japanese encephalitis virus [10], suid herpesvirus 1 [11], porcine circovirus type 2 [12], classical swine fever virus [13], *Toxoplasma gondii* [14], and *Salmonella enterica* [15]. MJ has also been used for the measurement of porcine C-reactive proteins as a method of monitoring health status [6]. However, the use of MJ for the detection of FMDV has not yet been characterized. We report on the feasibility of MJ as a sample matrix for the detection of FMDV by rRT-PCR, lateral flow immunoassay (LFI), and virus recovery through transfection of cultured cells using extracted viral RNA from MJ. The rRT-PCR provides evidence of FMDV RNA in MJ, while LFI confirms the presence of viral antigen. The presence of FMDV RNA was confirmed through VP1 sequencing and recovery of live FMDV by transfection of cultured cells with extracted RNA from MJ.

## 2. Results

### 2.1. Clinical Signs in Pigs

Three of the 6 pigs in each of 6 groups were each anesthetised with isoflurane before inoculation with 10^3^ tissue culture infectious dose 50 (TCID_50_) of FMDV A22 IRQ 24/64 (first experiment) or FMDV SAT2 ZIM 5/81 (second experiment) in the bulb of the left hind limb per pig. The rest of the pigs in each group were infected by direct contact with the directly inoculated pigs.

For both FMDV A22 IRQ 24/64 and FMDV SAT2 ZIM 5/81, clinical signs, including a slight increase in rectal temperatures, vesicles on the feet, and lameness, were seen in pigs starting at day post infection (DPI) 2–3. Disease progression in the pigs was as expected, with the directly inoculated pigs showing viremia and clinical signs 24–72 h prior to the direct contacts. Pigs with the most severe clinical signs were selected for euthanasia and tissue collection at scheduled time points. 

### 2.2. FMDV Detection in Meat Juice and Other Samples by Real-Time Reverse Transcription Polymerase Chain Reaction

Skeletal muscle (biceps femoris) was collected from animals experimentally infected with FMDV and MJ harvested after freeze-thaw cycles of skeletal muscle. RNA extractions were performed on MJ, serum, oral swabs, and tissue suspensions. Real-time reverse transcription polymerase chain reaction (rRT-PCR) was used to test the extracted RNA from these samples for the presence of FMDV genome.

#### 2.2.1. FMDV A22 IRQ 24/64 Experiment

**Figure 1:**
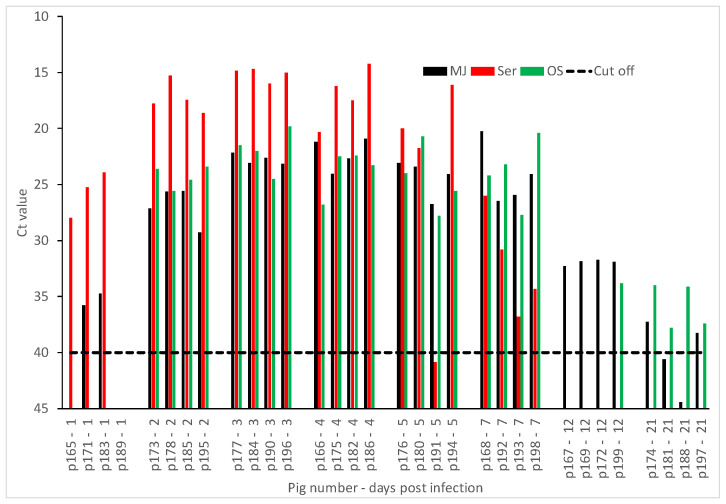
Detection of Foot-and-mouth disease virus (FMDV) in meat juice (MJ), serum (Ser), and oral swabs (OS) by rRT-PCR. Skeletal muscle (biceps femoris) was collected from animals experimentally infected with FMDV A22 IRQ 24/64 and MJ harvested after freeze-thaw cycles of skeletal muscle. Ribonucleic acid (RNA) was extracted from MJ, Ser, and OS collected at corresponding time points and tested by each rRT-PCR. The cut-off Ct value for the rRT-PCR is 40. Samples with Ct values less than 40 are considered positive for FMDV.

In the FMDV A22 IRQ 24/64 experiment, FMDV genome was detected in MJ as early as DPI 1 to as late as DPI 21 (Figure 1). Viremia based on FMDV RNA detection in sera started at DPI 1 (Figure 1) and was cleared within 4–5 days after first detection. FMDV RNA was also detected in oral swabs starting at DPI 2 (Figure 1) and was still detectable at 21 DPI in oral swabs. FMDV RNA was detected in MJ and oral swabs longer than in serum. The VP1 sequence of FMDV from MJ was >99% identical to the A22 IRQ 24/64 inoculum (data not shown).

**Figure 2:**
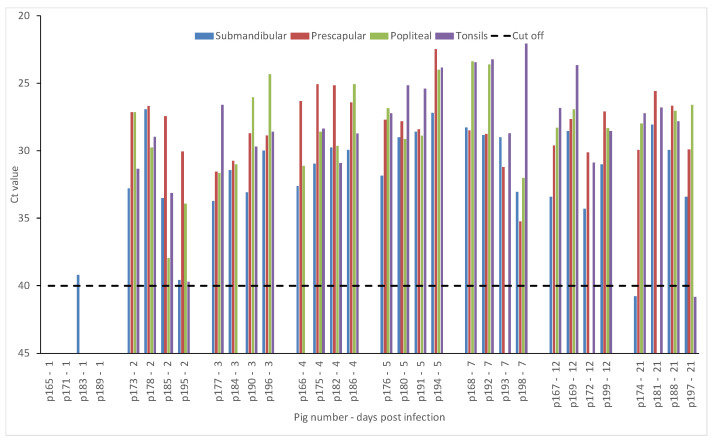
Crossing threshold (Ct) values for rRT-PCR for various tissue suspensions (submandibular lymph nodes, prescapular lymph nodes, popliteal lymph nodes, and tonsils) from FMDV A22 IRQ 24/64 infected animals. 10% tissue suspensions were prepared in PBS and clarified by centrifugation. RNA was extracted from tissue suspensions and tested by rRT-PCR. The cut-off Ct value for the rRT-PCR is 40. Samples with Ct values less than 40 are considered positive for FMDV.

FMDV RNA was detected in the submandibular lymph node from one animal at DPI 1. By DPI 2, all tested lymph nodes (submandibular, prescapular, and popliteal) and tonsils were positive for FMDV RNA and stayed positive up to 21 DPI (Figure 2).

#### 2.2.2. FMDV SAT2 ZIM 5/81 Experiment

**Figure 3:**
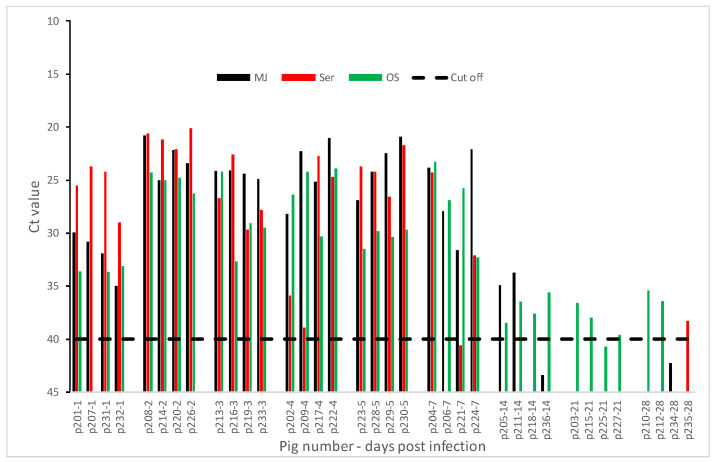
Detection of FMDV in meat juice (MJ), serum (Ser), and oral swabs (OS) by rRT-PCR. Skeletal muscle (biceps femoris) was collected from animals experimentally infected with FMDV SAT2 ZIM 5/81 and meat Juice (MJ) harvested after freeze-thaw cycles of skeletal muscle. RNA was extracted from MJ, Ser, and OS collected at corresponding time points and tested by each rRT-PCR. Cut off Ct value for the rRT-PCR is 40. Samples with Ct values less than 40 are considered positive for FMDV.

In the FMDV SAT2 ZIM 5/81 experiment, FMDV genome was detected in MJ as early as DPI 1 to as late as DPI 14 (Figure 3). A fragment of the VP1 sequence of FMDV from MJ was successfully determined and shown to be >99% identical to the SAT2 ZIM 5/81 inoculum (data not shown).

Viremia started at DPI 1 (Figure 3) and was cleared within 4–5 days after detection. FMDV RNA was detected in oral swabs at DPI 1–14.

**Figure 4:**
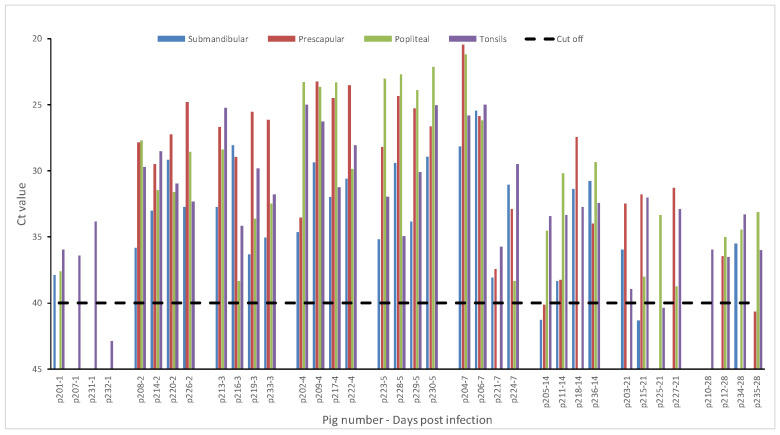
Crossing threshold (Ct) values for rRT-PCR for various tissue suspensions (submandibular lymph nodes, prescapular lymph nodes, popliteal lymph nodes, and tonsils) from FMDV SAT2 ZIM 5/81 infected animals. 10% tissue suspensions were prepared in PBS and clarified by centrifugation. RNA was extracted from tissue suspensions and tested by rRT-PCR. Cut off Ct value for the rRT-PCR is 40. Samples with Ct values less than 40 are considered positive for FMDV.

FMDV RNA was detected in the lymph nodes and tonsils starting at DPI 1. By DPI 2, all tested lymph nodes and tonsils were positive for FMDV RNA and stayed positive up to 28 DPI (Figure 4).

### 2.3. Antigen Detection by Lateral Flow Immunoassay

MJ samples from naïve and FMDV-infected pigs were tested for antigen detection by serotype-specific lateral flow immunoassay (LFI). Twenty-four MJ samples from naïve pigs were negative on both FMDV A22 IRQ 24/64 and SAT2 ZIM 5/81 LFIs (data not shown).

**Figure 5:**
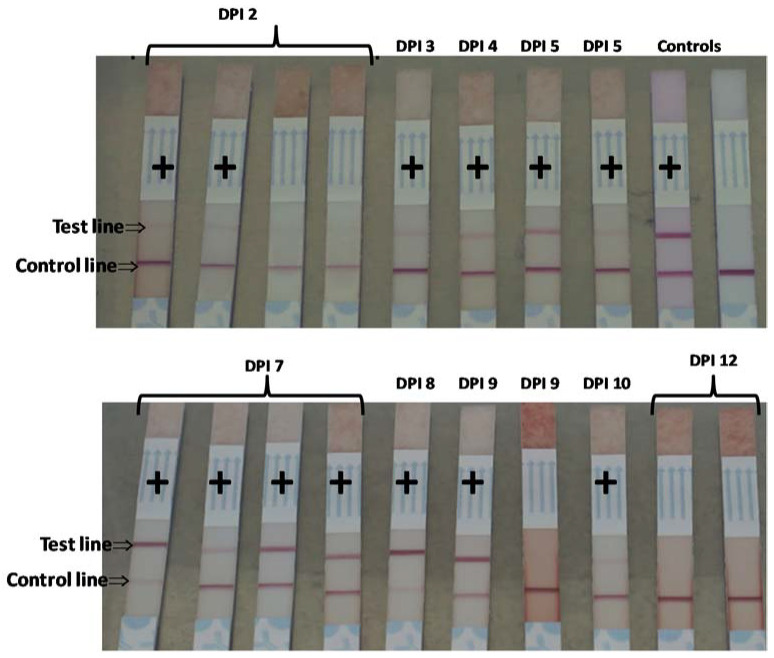
Detection of FMDV serotype A antigen in meat juice by lateral flow immunoassay. Meat juice was harvested from pigs inoculated with FMDV A22 IRQ 24/64 and tested for FMDV serotype A antigen by lateral flow immunoassay. + = positive for FMDV antigen, DPI = days post-infection.

With the FMDV A22 IRQ 24/64 experiment, FMDV antigen was detected in MJ at 3 DPI with faint test bands. These bands increased in intensity at DPI 5–9 (Figure 5).

**Figure 6:**
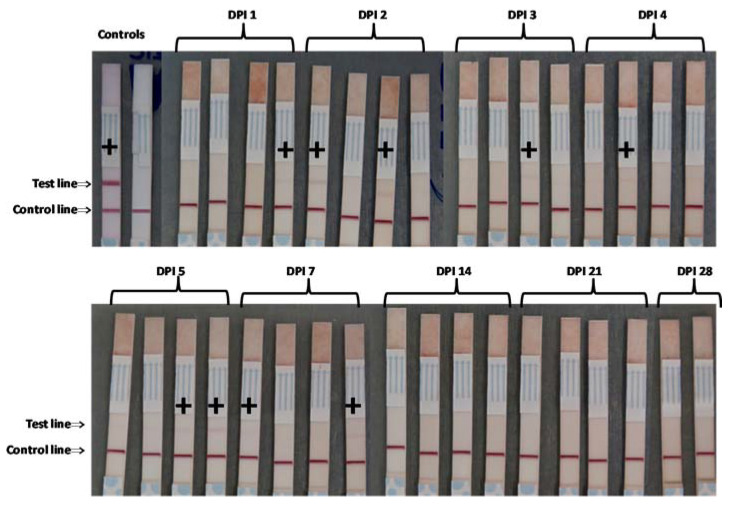
Detection of FMDV SAT 2 antigen in meat juice by lateral flow strip test. Meat juice was harvested from pigs inoculated with FMDV SAT2 ZIM 5/81 and tested for FMDV serotype SAT 2 antigen by lateral flow immunoassay. + = positive for FMDV antigen, DPI = days post-infection.

For the FMDV SAT2 ZIM 5/81 animal experiment, FMDV antigen was detected at 2–7 DPI, but the test bands were all faint compared to the positive control (Figure 6).

### 2.4. Virus Recovery from Nucleic Acid by Transfection

Virus isolation from MJ was attempted without success despite the presence of FMDV RNA as detected by rRT-PCR. In an effort to recover infectious material from rRT-PCR-positive MJ, susceptible cells were transfected with RNA from MJ. 

For the FMDV A22 IRQ 24/64 experiment, CPE was observed after 2 passages in LFBKαVβ6 cells for RNA from MJ samples collected at DPI 3, 7, and 12. CPE was observed after the first passage for the positive control.

For the FMDV SAT2 ZIM 5/81 experiment, CPE was observed on the first passage for RNA from MJ samples collected at DPI 2 and 5. CPE was also observed after the first passage for the positive control. The recovered virus was confirmed positive for FMDV by rRT-PCR and LFI.

## 3. Discussion

The primary objective of this study was to assess MJ as a sample type for FMDV detection in situations where commonly used samples are not available. To obtain these MJ samples, pigs were experimentally inoculated with FMDV. Our data shows that FMDV can be detected in MJ primarily by rRT-PCR. In addition, FMDV antigen can be detected in MJ at specific time points using LFI. FMDV could not be isolated from MJ, but infectious material could be recovered by the transfection of LFBKαVβ6 cells with nucleic acids from MJ. Failure to isolate FMDV from MJ could be attributed to virus inactivation by lactic acid. Indeed, MJ had a pH of 6. MJ is inherently acidic due to lactic acid build-up caused by rigor mortis. FMDV is inactivated in muscles from acid buildup [16]. The acid causes degradation in the protein structure of the FMDV capsid. Without proper adhesion molecules, viral attachment is not possible for delivery of the FMDV genome into susceptible cells. Freezing of samples temporarily suspends acid formation until thawing [17]. However, once tissue samples are thawed, acid formation proceeds and inactivates any virus present [17]. In these acidic conditions, RNA has been shown to remain stable while DNA becomes degraded through depurination [18].

Based on the rRT-PCR results, MJ and serum samples from the same pigs were positive for FMDV RNA from DPI 2–7. It can be assumed that the presence of a virus in the blood implies that it will most likely be present in muscles since blood flows into the latter. This would probably be true for the early time points post-infection. However, even in the absence of viremia, MJ samples from DPI 12–21 were positive for FMDV genome. It is possible that the clearance of the virus from muscles happens at a slower rate than from blood, possibly because the virus is located within the myocytes or adipocytes and is only released when cells are disrupted by freeze-thawing. Attempts to locate FMDV within the muscles by immunohistochemistry and in situ hybridization were unsuccessful. The second possibility is that the virus could have originated from the regional lymph nodes and be released into muscles through the lymphatic system. FMDV is known to persist in popliteal lymph nodes for more than 28 days post-infection [19]. We also showed that lymph nodes from infected pigs were positive for FMDV RNA at the end of the experiments at DPI 21 and 28 for FMDV A and SAT2, respectively.

Detection of FMDV antigen in MJ by LFIs shows great potential for use of LFI as a point of need test in circumstances of illegal importation of meat. However, antigen detection by LFI was short-lived compared to rRT-PCR, implying that LFI might fail to detect low levels of viral antigen in MJ. rRT-PCR is inherently a more sensitive assay [20] and thus explains the detection of FMDV RNA over a wider duration of the experiment. The fact that LFI can detect FMDV in MJ is a good reason to collect both MJ and sera when possible, considering that FMDV antigen detection in serum samples by LFIs has not been successful [21]. 

In some circumstances, virus isolation from clinical samples might be necessary for downstream analysis. However, virus isolation from MJ samples was attempted without success. Transfection is an alternative rescue system where the viral genomic RNA can be transfected into susceptible cell lines to allow replication of positive-sense RNA and production of progeny virus. Previous research confirmed the ability to recover FMDV by transfection [22,23]. Through transfection with Lipofectamine 3000, FMDV was recovered from RNA extracted from vesicular material stored at room temperature over three weeks [22]. This model was used to recover FMDV from MJ and proved that infectious FMDV can be obtained from MJ when FMDV RNA is present in the tissues.

The presence of FMDV RNA in MJ was further confirmed by sequencing of the VP1 gene of FMDV. Consensus sequences from four different MJ samples showed >98% similarity to the FMDV inoculum that was used to infect the pigs. 

In conclusion, MJ is a useful sample for FMDV detection alongside traditional sample types and in exceptional circumstances when the usual samples are not available. The virus might be rapidly inactivated in MJ, but infectious virus is recoverable by transfection of FMDV RNA into susceptible cells. The prolonged detection of FMDV RNA in MJ demonstrates the additional benefit of using this sample type, in addition to any available conventional samples, for rRT-PCR to test for FMDV.

## 4. Materials and Methods

### 4.1. Viruses

FMDV Serotype A (A22 IRQ 24/64) and Southern African Territories 2 (SAT2 ZIM 5/81) were used in separate studies. The viruses were obtained from the World Reference Lab for FMDV, Pirbright Institute. Each virus was amplified in baby hamster kidney 21 (BHK-21) cells, the cell culture supernatant harvested after cytopathic effect (CPE) was observed, and the virus titre determined as previously described [24].

### 4.2. Experimental Animals and Inoculations

The Animal Care Committee at the Canadian Science Centre for Human and Animal Health approved this study under the Animal Use Document (AUD) number C-18-004. Thirty-six pigs at 5-6 weeks old were used for each FMDV serotype (A and SAT2). The pigs were purchased locally from Manitoba farmers and transported to the laboratory following the Canadian Council on Animal Care guidelines. These pigs were separated into six groups of six pigs and each group was housed together in a cubicle. The animals were given one week to acclimate to the environment prior to inoculation with the virus. Feed was provided twice daily and water was provided ad libitum.

#### 4.2.1. Animal Inoculations

FMDV inoculum was prepared by diluting the virus to 5 × 10^3^ tissue culture infectious dose 50 (TCID50)/mL in cell culture media. Three of the six pigs in each cubicle were each anesthetised with isoflurane before inoculation with 0.1 mL of inoculum in each heel bulb of the left hind limb for a total of 0.2 mL of inoculum per pig.

#### 4.2.2. Monitoring and Sample Collection

Animals were monitored daily for clinical signs and their rectal temperature recorded. The lameness scoring system by Main et al. [25] and modified by Kilbride et al. [26] was used where 0 is normal and 5 is no movement. Blood, orals swabs, and oral fluid collection was done once before inoculation and once-daily post-inoculation from day 1–7, 12 or 14, 21, and 28 or when an animal reached the humane endpoint.

Ten millilitres of blood were collected from the anterior vena cava with a 20-gauge needle into BD Vacutainer Blood Collection SST Serum Separation Tubes (B367988, Becton, Dickinson and Company, Franklin Lakes, NJ, USA). Tubes were centrifuged at 3000× *g* for 20 min, 4 °C for separation of serum. Oral swabs were taken with Dacron tipped swabs and stored in 1 mL of BD Universal Viral Transport Media (Becton, Dickinson, and Company). Four pigs were euthanized each day from days 1–5, 7, 12 or 14, 21, and 28. Animals with clinical signs and/or animals that reached humane endpoints were euthanized first. Shortly after euthanasia, approximately 2 cm^2^ of muscle tissue from biceps femoris were collected and stored in re-sealable plastic bags. In addition, approximately 2 g of tonsil, submandibular lymph nodes, prescapular lymph nodes, and popliteal lymph nodes were stored in tubes containing 3 mL of Universal Transport Media (UTM). All samples were kept frozen at −70 °C. 

### 4.3. Meat Juice and Tissue Processing

Muscle tissue in a re-sealable plastic bag was removed from −70 °C and allowed to thaw at room temperature. Liquid exudates from the thawed muscle (MJ) released into the plastic bag were harvested with a sterile pipette and transferred into cryovials. Then, 10% tissue suspensions of the lymph nodes and tonsils were prepared using Precellys Lysing Kits (BER-P000918LYSK0A0, ESBE Scientific, Markham, ON, USA) as previously described [27,28]. MJ and tissue suspensions were stored at −70 °C until they were used for testing.

### 4.4. Real-Time Reverse Transcription PCR

RNA extractions were performed on MJ, serum, oral swabs, and tissue suspensions using the Applied Biosystems MagMAX-96 Viral RNA Isolation Kit (AMB1836-5, Life Technologies, Burlington, ON, USA) together with a MagMAX Express-96 Deep Well Magnetic Particle Processor (Life Technologies) as described previously [28]. 

The commercial Tetracore VetAlert™ FMDV RNA Test Kit (Tetracore Inc., Rockville, MD, USA) real-time reverse transcription polymerase chain reaction (rRT-PCR) was used to test the extracted RNA from various samples for the presence of FMDV genome. The assay was performed according to the manufacturer’s instructions using the Applied Biosystems 7500 Real-Time PCR System (4351106, Life Technologies). Crossing threshold (Ct) values less than 40 were considered positive.

### 4.5. Antigen Detection by Lateral Flow Immunoassay (LFI)

Meat juice samples from naïve and FMDV–infected pigs were tested for antigen by LFI for FMDV serotype A [21] and FMDV serotype SAT 2 [20] following the published protocols for each serotype. Each test utilizes two conjugated serotype-specific monoclonal antibodies. One antibody is conjugated to biotin, which is captured at the test line by streptavidin, while the other antibody is conjugated to gold for visualization of bound antigen. Limit of detection for the test is 10^3.77^ TCID_50_ for serotype SAT2 and 10^5.50^ for serotype A [20,21].

### 4.6. Virus Recovery from Nucleic Acid by Transfection

Transfection reagents (Lipofectamine 3000, L3000001, Invitrogen, Carlsbad, CA, USA) were prepared according to the manufacturer’s protocol. Briefly, Lipofectamine 3000 reagent (Invitrogen) was mixed with Opti-MEM (31985-062, Gibco, Life Technologies) as instructed in the kit protocol. RNA extracted from MJ, as described above, was diluted in Opti-MEM and then mixed with P3000 reagent. The RNA and P3000 mixture was then mixed with the Lipofectamine 3000 reagent, which was previously diluted in Opti-MEM, and incubated at room temperature for 15 min. During the incubation period, 90% confluent LFBKαVB6 cells on a 24 well plate was prepared for transfection by aspirating cell culture supernatant, followed by a wash of the cell monolayer with pre-warmed sterile PBS (311-425-CL, Wisent Inc.). PBS was aspirated and discarded, followed by another wash with supplemented DMEM. The RNA plus P3000 mixture was applied to the cells and then incubated at 37 °C for 15 min. Following incubation, each well was topped up with supplemented DMEM containing 2% FBS and then incubated at 37 °C for approximately 48–72 h. The plates were monitored daily for CPE. RNA from FMDV A22 IRQ 24/64 and SAT2 ZIM 5/81 (amplified in cell culture) were used as positive controls while PBS was used as a negative control.

A second passage was performed by collecting supernatant from each well after freeze-thawing cycles and centrifugation to pellet cells. Then, the supernatant was transferred onto a new plate of LFBK cells. The plate was incubated for 48–72 h and monitored for CPE progression.

### 4.7. Sequencing of FMDV RNA from Meat Juice Samples

RNA was extracted from MJ samples as described previously [28]. The VP1 region of FMDV was amplified using the qScript One-Step RT-qPCR Kit and VP1 specific primers for each serotype of FMDV. The PCR reactions were run on an ABI GeneAmp PCR System 9700 using the following cycling conditions: Stage 1 (48 °C for 20 min), Stage 2 (94 °C for 3 min), and Stage 3 (40 cycles of 94 °C for 20 s, 55 °C for 30 s, 68 °C for 1.5 min). The PCR products were purified using the QIAGEN PCR Purification Kit, followed by de novo sequencing using the BigDye Terminator v3.1 Cycle Sequencing Kit performed on the ABI 3500xL Genetic Analyzer.
